# Barriers and Facilitators in the Implementation of Digital, Blended, and Analog Evidence-Based Osteoarthritis Care: International Qualitative Expert Interview Study

**DOI:** 10.2196/85941

**Published:** 2026-10-23

**Authors:** Theresa Sophie Busse, Julia Nitsche, Ina Carola Otte, Nino Chikhradze, Barbara Jömann, Horst Christian Vollmar, Jennifer Bosompem

**Affiliations:** 1 Digital Health, Department of Human Medicine, Faculty of Health, Witten/Herdecke University, Alfred-Herrhausen-Str. 50, Witten, 58455, Germany, 0049 2302 926 ext 708; 2 Institute of General Practice and Family Medicine (AM RUB), Medical Faculty, Ruhr University Bochum, Bochum, Germany; 3 Didactics and Educational Research in Health Science, Department of Human Medicine, Faculty of Health, Witten/Herdecke University, Witten, Germany; 4 Health Services Research, Institute for Diversity Medicine, Medical Faculty, Ruhr University Bochum, Bochum, Germany; 5 Institute for Diversity Medicine, Medical Faculty, Ruhr University Bochum, Bochum, Germany

**Keywords:** physiotherapy, blended care, implementation science, osteoarthritis, osteoarthrosis, musculoskeletal diseases, conservative treatment

## Abstract

**Background:**

Evidence-based nonsurgical osteoarthritis (OA) care programs improve patient outcomes and may reduce the burden of disease. Despite their demonstrated effectiveness, their implementation into routine health care remains limited across health care systems. Understanding factors that facilitate or hinder implementation is essential to support broader adoption and long-term sustainability worldwide.

**Objective:**

This study aimed to explore factors that facilitate or hinder the implementation of evidence-based nonsurgical OA care programs. Specifically, it examined implementation across different health care settings, implementation phases, and analog, digital, and blended care models to identify transferable insights for implementation in different national and regional contexts.

**Methods:**

We conducted a qualitative expert interview study with 12 international experts involved in evidence-based OA care programs implemented across 6 countries. Semistructured interviews explored experiences with program development, implementation, and further development of analog, digital, and blended care models. Interview transcripts were analyzed using qualitative content analysis according to Kuckartz, using deductive and inductive category development.

**Results:**

Key barriers included limited funding and reimbursement structures, insufficient political support, organizational complexity, and legal or regulatory requirements. Important facilitators comprised strong leadership, interdisciplinary collaboration, structured communication, adaptation to local health care contexts, and the use of existing international program structures. Participants further emphasized that implementation requirements varied across the preparation, initial implementation, and consolidation phases, highlighting the need for phase-specific implementation strategies.

**Conclusions:**

Successful implementation of evidence-based nonsurgical OA care programs requires sustainable funding, stakeholder engagement, context-specific adaptation, and structured implementation planning across all implementation phases. Existing international program structures and collaboration may facilitate implementation across health care systems while reducing development efforts. Future implementation initiatives should consider phase-specific requirements and local contextual factors to improve long-term adoption and sustainability.

## Introduction

Prevention and health promotion approaches are becoming increasingly important in health care. One area in which such approaches have shown promising results is the management of knee and hip osteoarthritis (OA). This musculoskeletal disease affects up to 40% of people older than 65 years [1], leads to high costs [2], and causes a burden on patients.

For mild to moderate OA, guideline-recommended treatment is nonsurgical and combines patient education, exercises, and weight reduction [3-5]. These components are increasingly delivered through structured OA treatment programs, spanning analog, digital, and blended care models implemented at regional, national, and international levels. Such programs may be developed specifically for OA or adapted from interventions targeting other musculoskeletal conditions. Evidence suggests that these approaches are safe, effective, cost-efficient, and suitable for routine care [3].

Despite growing evidence and international interest, the integration of OA programs into routine health care remains limited [6]. Consequently, patient access outside research settings is often restricted [6,7]. The limited uptake reflects the continuing challenge of translating research findings into clinical practice [4,7].

Despite growing evidence supporting OA care programs, important knowledge gaps remain regarding how these interventions can be successfully implemented across different health care systems, delivery models, and implementation stages. To address this gap, we conducted qualitative interviews with experts involved in established OA care programs. The study addresses the following research question: What factors influence the implementation of evidence-based OA treatment programs across different health care settings, implementation stages, delivery formats (digital, analog, and blended), and stakeholder groups?

We explored barriers and facilitators to implementation across health care settings, implementation stages, delivery formats, and stakeholder groups.

By synthesizing expert perspectives across multiple international programs, this study aims to provide practice-oriented insights into implementing evidence-based OA care in routine practice and contributes to the broader implementation science literature [8].

## Methods

The reporting of the study results follows the COREQ (Consolidated Criteria for Reporting Qualitative Research) framework [5].

### Study Design

The qualitative approach was chosen due to its suitability for exploring contextual and implementation-related factors that cannot be fully captured through quantitative methods. We conducted semistructured interviews with experts in the field of treatment programs for knee and hip OA as well as back pain.

To identify relevant programs, we first carried out a rapid review focusing on conservative treatment programs for knee and hip OA [7]. Based on this rapid review, we assembled a list of existing approaches and consulted international experts to gain insights into national characteristics and contextual influences.

In addition to OA programs, the review identified one back pain program that had been developed through adaptation of an established OA program. We included this program because it represented a unique example of how implementation strategies and program structures can be transferred across musculoskeletal conditions. Its inclusion enabled exploration of implementation processes beyond a single diagnosis while maintaining a focus on comparable nonsurgical, exercise-based care models.

The selected interventions additionally provided insights into how treatment concepts have been adapted across countries and how care models differ between analog, digital, and blended care [9].

### Program Overview

The included programs differed in their form of delivery, implementation strategies, and national contexts. Better Management of Patients with OsteoArthritis served as the foundation for the subsequent development of GLA:D (Good Life with osteoArthritis in Denmark) [10,11], while Joint Academy emerged as a digital adaptation designed to improve accessibility and overcome barriers to participation in conventional OA care [12]. Studies accompanying the GLA:D program showed a significant reduction in the risk of being unable to work due to knee or hip OA, as well as improvements in pain intensity, physical resilience, and everyday activity [6,11].

Although differing in form of delivery and organizational structure, the programs shared a common evidence-based treatment philosophy.

### Recruitment and Participants

JB and TSB designed the recruitment strategy to target participants with specific expertise in implementing OA treatment programs for knee and hip. Eligible participants were professionals who had been directly involved in the development, coordination, implementation, or scientific evaluation of one of the identified programs. The interviewers (JB and TSB) had no prior professional relationship with the participants before recruitment except for one participant, with whom they had had occasional contact during a joint project in the past. This did not affect the interview situation.

The participants were informed that both researchers are currently working on the facilitating and inhibiting factors in implementation research and about the insights they had gained from the rapid review mentioned above.

In the beginning, JB and TSB compiled a list of all OA treatment programs for knee and hip that had been identified worldwide in the previously mentioned review. JB and TSB then identified and contacted the program coordinators responsible for the development and implementation of these programs. In addition, they expanded recruitment through expert recommendations provided by interview participants (snowball sampling). Recruitment continued until additional interviews were considered unlikely to provide substantially new insights regarding the implementation processes under investigation.

The research team invited all identified candidates via email to participate in the study. Candidates were asked to complete a short questionnaire regarding sociodemographic information.

In total, the research team contacted 30 experts and one additional contact email address for a specific treatment program. Twelve experts agreed to participate in the study. Recruitment and interviews were conducted between August and October 2023. The final sample was considered appropriate because it captured experts from multiple countries, health care settings, and implementation roles, providing sufficient variation to address the study aim.

### Data Collection

Interviews were conducted in English or German, depending on the interviewee’s language preference. Accordingly, we developed the interview guides in both languages and ensured full content equivalence across both versions.

The interview guides were reviewed by an orthopedic surgeon for technical adequacy and by a team of research assistants from the Institute of General Practice and Family Medicine at Ruhr University Bochum during a qualitative research workshop. This review process focused on content relevance, comprehensibility, and alignment with the study objectives. Revisions were made iteratively based on the feedback received and discussed within the group of authors.

The semistructured interview guides (see Multimedia Appendix 1) focused on 4 key thematic areas:

1. Background information about the treatment program and the expert’s role within it;
2. Description of the implementation process;
3. Experiences in program development since implementation; and
4. Considerations, expectations, and wishes for future development of the program.

Interviews were conducted by 2 female researchers (TSB: doctoral degree in health sciences; JB: MSc in health economics), both trained in qualitative research and employed at the Institute of General Practice and Family Medicine at Ruhr University Bochum at the time of the study. In most cases, both researchers attended the interviews—one leading the interview and the other monitoring the process and taking notes. TSB and JB took turns in this role to ensure consistent quality.

Neither interviewer was involved in the development or implementation of any of the included OA treatment programs. This helped to reduce potential conflicts of interest and enabled participants to discuss implementation experiences from an external perspective. Throughout data collection, the interviewers documented contextual observations and reflected on potential influences on the interview process in field notes.

All interviews were conducted via videoconferencing (using Zoom by Zoom Communications), audio-recorded, pseudonymized, and transcribed by a professional transcription service. German interviews were transcribed verbatim in German, just as English interviews were transcribed verbatim in English. The interviews were always analyzed in the original language. German quotations selected for publication were translated into English by the research team, and the translations were checked by 2 different authors.

In one case, the formal interview was preceded by a detailed preliminary conversation, which the participant had requested before agreeing to the interview. During this conversation, the participant described his role in the program. The participant consented to audio recording only after this preliminary exchange and had limited time available. To avoid redundancy and respect these constraints, the thematic first block of the interview guide was omitted during the recording. This interview was still included in the analysis, as 3 researchers (JB, JN, and TSB) independently agreed that the data provided sufficient contextual richness. The preliminary conversation was not included as primary interview data.

### Data Analysis

We analyzed the interview transcripts using qualitative content analysis following the approach described by Kuckartz [13]. Interviews were transcribed shortly after completion of data collection and prepared for analysis. The formal coding and category development were conducted between September and November 2024 using MAXQDA software by VERBI GmbH (TSB: MAXQDA 2024; JN: MAXQDA 2022). The time lapse between data collection and analysis is attributable to the authors’ new professional positions and the subsequent conclusion of the project, which prevented them from working on the project earlier, and is not due to changes in the dataset. Coding was performed in English. Two researchers (TSB and JN), both holding doctoral degrees in health care science and trained in qualitative research, independently coded all interviews. Discrepancies were discussed during regular analytical meetings. Where differences in interpretation occurred, both researchers revisited the original transcript segments and agreed on a final coding decision through discussion and consensus.

We applied a structured content-analytic approach combining deductive and inductive category development. Initial main categories were derived deductively from the interview guide, while additional categories were generated inductively from the data. Throughout the analysis, the coding framework was iteratively refined and continuously compared across interviews to ensure analytical consistency.

To enhance credibility, a third researcher (JB) with experience in qualitative content analysis reviewed the final category system and code tree for plausibility, coherence, and completeness. Feedback from this review informed the final interpretation and refinement of the category system, which was finalized in March 2025.

Methodological rigor was supported through independent coding, regular consensus meetings, and review of the final category system by a third researcher. Field notes were used to contextualize interview data and support interpretation.

### Ethical Considerations

The study received a favorable ethics vote from the Ethics Committee of Ruhr University Bochum (register number 21-7438) in February 2022.

Interested individuals received detailed study information outlining the study’s objectives, scope, and participation requirements. To formally participate, candidates were required to sign a declaration of consent. Participants returned the signed consent forms via email.

Participation in the interviews was voluntary. Participants could at any time—even without giving a reason—decline to participate or withdraw their consent and leave the study without suffering any adverse consequences as a result.

Participants did not receive compensation for the interviews.

## Results

### Participant Overview

The recruitment process resulted in 12 completed interviews with experts involved in 8 different treatment programs. The interviews ranged from 33.42 to 65.02 minutes, with an average duration of 49.5 (SD 9.42) minutes.

The majority of experts (8 of 12) were affiliated with the GLA:D network, making it the most represented program in the sample. Specifically, experts participated from GLA:D country programs in Germany (n=2), New Zealand (n=2), Switzerland (n=2), Canada (n=1), and Denmark (n=1). Another expert from the Danish GLA:D Back program (n=1) took part in an interview. In addition, experts from the Joint Academy Program (n=2) and the Better Management of Patients with OsteoArthritis program in Sweden (n=1) were interviewed.

Figure 1 provides an overview of the programs in which the experts were involved, detailing their structural characteristics and the national context in which the interviewed experts had worked.

The age of participants as of 2024 ranged from 35 to 81 years, with an average age of 53.4 years (SD: 13.35). Three experts identified as female and 9 as male. To protect the anonymity of the participants, no further sociodemographic data are reported in this publication.

**Figure 1.**
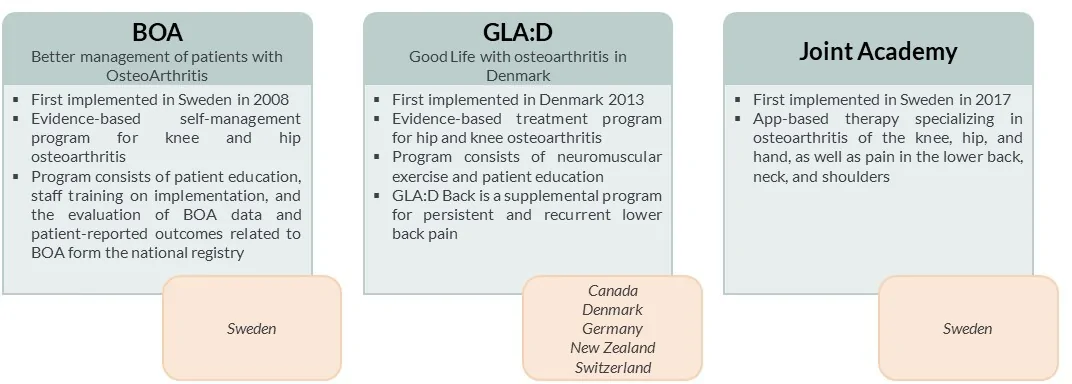
Overview of associated osteoarthritis treatment programs (own representation). BOA: Better Management of Patients with OsteoArthritis; GLA:D: Good Life with osteoArthritis in Denmark.

### Results of the Data Extraction

#### Category System

During the analysis, the research team assigned 1043 codes to 7 main categories covering contextual information on care provision before implementation, interviewee and program characteristics, implementation processes, program development, and overarching reflections on OA care.

A detailed overview of the category system (first level) is provided in Figure 2. Although the overall coding combined deductive and inductive category development, the 3 implementation phases emerged inductively from the interview data and were subsequently used to structure the presentation of the findings.

A detailed overview of the category system (first level) is provided in Figure 2. Although the overall coding combined deductive and inductive category development, the 3 implementation phases emerged inductively from the interview data and were subsequently used to structure the presentation of the findings.

**Figure 2.**
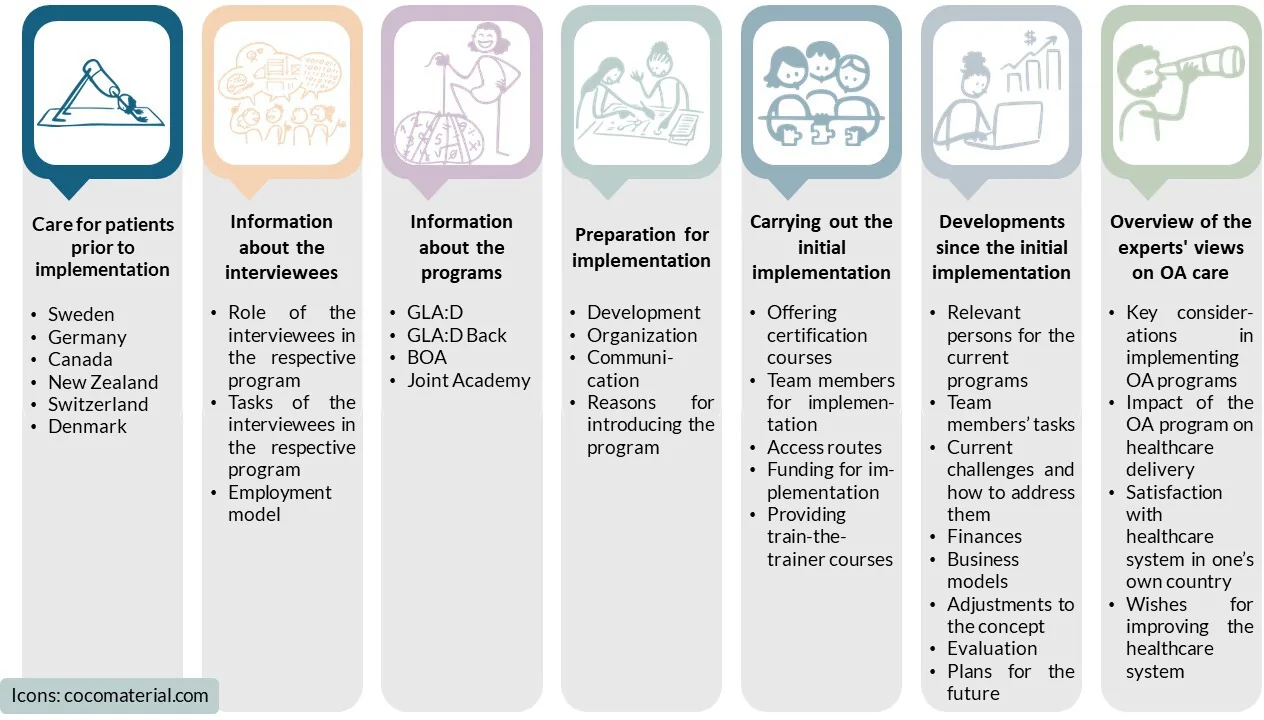
Main categories and subcategories of the qualitative analysis (own representation). Icons sourced from Coco Material [14] published under CC0 1.0 Universal license [15]. BOA: Better Management of Patients with OsteoArthritis; GLA:D: Good Life with osteoArthritis in Denmark.

Across programs and countries, experts described implementation as a dynamic process consisting of 3 interrelated phases: preparation, early implementation, and consolidation. Although individual programs differed in their design and organizational context, similar implementation challenges and enabling factors emerged across settings. The following sections present the findings according to these implementation phases.

These implementation phases provide the framework for the presentation of the results in the following sections. Despite variations in program design (see Figure 1), a collective reporting approach addresses the research questions comprehensively.

#### Preparation and Development

##### Reasons for Introducing the Program

Across programs, implementation was primarily motivated by perceived deficiencies in existing OA care pathways. Experts described the introduction of OA programs as an opportunity to improve access to evidence-based treatment, reduce regional inequalities, and standardize care delivery. Established implementation models, particularly GLA:D, frequently served as inspiration and provided practical guidance for local adaptation.

*The main goal is to give a more equal healthcare to patients with OA in [Country]. Because we knew back in the days it was a little bit different depending on where you're living if you're getting an education, a training or not. So, I think equality was the first goal*.

[Interviewee 9]

Experts reported that, in several programs, the COVID-19 pandemic accelerated the adoption or expansion of digital and blended forms of delivery and that these models were subsequently valued for their scalability and potential to increase access to care.

##### Adaptations and Development

The development process ranged from entirely new programs to adaptations of evidence-based programs. One program, for example, began as a small university pilot that underwent multiple iterations before public rollout. Development tasks included designing analog materials (eg, screening tools, educational content, training modules, preparation materials) and digital components for blended care, as well as setting up data registries.

With the help of train-the-trainer models, therapists were able to deliver the program independently.

In the case of GLA:D adaptations, the Danish team was consulted for linguistic and cultural fit. One interviewee advised building on existing programs:

*So don't reinvent the wheel yourselves. Even though you might not be happy with all parts of it, I would really encourage you to take on something that is already developed and structured*.

[Interviewee 10]

Program adjustments considered country-specific conditions and multicultural environments. Cost-effective equipment, intensive simple training units, and well-developed educational concepts were also highlighted as helpful elements.

##### Initial Team and Actors Involved

During preparation, work was mainly driven by small core teams consisting of research managers, developers, or project leads. Legal advisors, patients, and therapists provided feedback. Experts emphasized that practitioners from the relevant field should be involved to ensure practical understanding:

*But you do need to have someone driving it who has a really good understanding of the research and understanding of how that works, the business model, but also the whole training program. [...] And don't get them to do it for free*.

[Interviewee 2]

##### Organizational Tasks

Organizational efforts involved contracts, bureaucracy, university resource allocation, and securing legal permissions, as well as GLA:D license agreements with Danish developers for data transfer.

*Plus, in the [Country] healthcare context, in the vast majority of cases, universities do not have the resources to enter a program, no matter how good it is, into the healthcare sector*.

[Interviewee 5]

##### Financing

Experts emphasized that securing sustainable financing for program development and initial establishment was a prerequisite for implementation. While funding models differed between countries and health care systems, experts consistently emphasized the importance of reimbursement mechanisms, institutional support, and early engagement with payers. Reliance on short-term project funding was widely viewed as insufficient for long-term implementation.

*And because we have had good experiences in [Country] with the 18 units that we bill and charge to the health insurance companies, we have decided to adopt this structure identically, but this has meant that we first carry out four times two exercise units in the first four weeks, followed by four times one exercise unit, so that in the end we also have a total of 18 units, which we then bill to the health insurance companies and payers*.

[Interviewee 11]

##### Communication

Communication activities focused on building legitimacy and awareness among key stakeholders. Experts highlighted the importance of engaging professional associations, health care providers, patient representatives, and policymakers early in the process. Public visibility through media appearances, conferences, and awareness campaigns was perceived as an important facilitator of implementation.

*So I think, looking back (...) the kick-off was really great for us with this TV appearance, even if it took an incredible amount of time and energy to get everything off the ground*.

[Interviewee 1]

### Carrying Out the Initial Implementation

During rollout, responsibilities expanded to include trained therapists (chiropractors, physiotherapists, physicians, and nurses), scientists, software developers, and regional coordinators. Interviewees emphasized the importance of creating local ownership among clinicians, who frequently acted as ambassadors and facilitators within their professional networks:

*And on the first, I would say, five, ten courses we were also very focused on ensuring that the new clinicians that had received the course were also ambassadors for the program in their local community*.

[Interviewee 12]

Rollouts varied from regional pilots scaling nationally to single-insurer collaborations or multiregion launches.

Initial funding during the rollout phase came from associations, grants, or public sources, though high license fees and missing reimbursements hindered progress.

### Consolidation and Ongoing Development

#### Members of the Program Team and Actors Involved

After implementation, operational responsibility increasingly shifted from the original developers to coordinators, database managers, analysts, and regional contacts. Initial developers stepped back as physiotherapists and coordinators took the operational lead.

*But then gradually their role was less and less, [...] in the early days they were really important*.

[Interviewee 12]

The recruitment of therapists emphasized openness without pressure.

*So it’s usually not so difficult to get a physiotherapist to try. But don't try to convince anyone [...]*

[Interviewee 8]

#### Further Development of the Program

The teams initiated an exchange with physicians on points of criticism regarding the program and regular strategy meetings of the team. Annual reports and health services research supported this process. They facilitated active exchange with patients, providers, health policymakers, and payers.

#### Feedback From Patients and Providers

Following implementation, experts reported generally positive feedback from both patients and providers. Perceived benefits included improved access to evidence-based care, greater standardization of treatment practices, enhanced professional knowledge among participating clinicians, and low equipment requirements. However, providers also identified barriers related to workload, reimbursement structures, organizational demands (lack of experience with group training and duplication of documentation), and the practical challenges of delivering group-based interventions.

*But we're getting more and more orthopedic surgeons who see the value of conservative management [...]*

[Interviewee 2]

#### Adjustments Based on the Feedback and Research Findings

Following feedback and research findings, the teams introduced quality assurance measures such as recertification, knowledge checks, and additional support services (eg, support team, coaching, webinars, and information materials), refined program content, revised didactics, and adapted teaching materials.

Some interviewees nevertheless reported a lack of support during implementation.

#### Organizational Tasks

Team tasks included coordination, organizing, and ensuring data collection. Programs that established registries documented a wide range of clinical parameters at program entry and exit, enabling pre-post comparisons and cross-regional and cross-national analysis. The data ensure quality and help to demonstrate effectiveness to policymakers. In addition, programs with digital components supported adherence monitoring for both educational content and exercise sessions.

#### Financing

After implementation, financial issues primarily concerned long-term sustainability for registries, quality assurance, administration, and stable reimbursement. The interviewees reported that while therapists faced only minimal costs (eg, for room rental or digital access and the initial train-the-trainer course), initiators bore expenses for the registry, digital design, and administration. It was possible to cover costs through research projects, risk capital, private investments, or self-funding by patients. Payment models varied widely, including national coverage, regional schemes, private funding, coverage by health insurance, or mixed models. Interviewees emphasized the perceived legitimacy of the program:

*Because if it’s available publicly, people think it’s got to work better. So, they will pay privately. But if it’s not available publicly they go: ‘Okay, obviously it’s not a good thing.’*

[Interviewee 3]

It was mentioned that no funding had been available for many years. To improve financing, interviewees recommended integrating the programs into the public health care system, negotiating with professional associations and health insurance companies, and conducting evaluations to demonstrate effectiveness. Positive developments were underway in some regions:

*We were probably a year too early with trying to launch the program, because there’s been significant funding change and now there is funding for osteoarthritis programs developed by physios from the state and the government*.

[Interviewee 3]

#### Perceived Limitations

Interviewees pointed out that the current structure might meet the needs of highly active patients and that programs lacked long-term motivation methods since lifelong training should be supported. One interviewee reported that personal trainers were introduced to bridge this gap.

#### Perceived Effects on Care

Experts reported a variety of factors that influence the impact of the respective programs on the care of patients with OA. They highlighted the international exchange for continuous improvement, changes in perceptions of conservative and surgical care, as well as earlier, more consistent, or even initial treatment, and positive treatment outcomes.

*And it’s not such a big benefit for the few who already provide very, very good care. [...] But I think there are many who provide care that is far from the quality standard [...]*

[Interviewee 6]

Experts perceived that the programs contributed to earlier access to conservative treatment, greater consistency in OA management, and improved patient outcomes. Several interviewees also reported that patients appeared better prepared when surgery eventually became necessary.

*[...] it’s very important that the patients are in the best possible shape [...] because that will make rehabilitation after surgery better and easier and they will reach a better result of the surgery as well*.

[Interviewee 7]

Interviewees underlined the value of group treatment in addressing the shortage of skilled workers and praised the low costs and accessibility of digitally designed programs, especially for patients for whom analog services are not attractive. They additionally pointed out their relevance for highly educated patients, those with multiple comorbidities, those living in urban areas with limited access to programs, as well as those living in rural areas with the same problem. This also has advantages for therapists in view of increased workplace flexibility:

*And there is a lot of young physiotherapists that like not to be in the office every day in the week [...]. Or you can make your own schedule and still get things going*.

[Interviewee 8]

### A Glimpse Into the Future

Despite perceived successes, experts identified several ongoing challenges, including insufficient reimbursement structures, continued reliance on surgical care pathways, limited implementation of evidence-based recommendations, and administrative burdens. To address these issues, interviewees emphasized the need for stronger political support, expanded digital services, improved professional education, and sustainable financing models.

*We need to replace some of our traditional ways with digital ways which are smarter, which are faster, which are lower cost to the system and the patient. And we need to find smarter ways and other ways, many of them digital, to find the resources without getting more money*.

[Interviewee 7]

Future priorities included increasing public visibility, strengthening quality assurance mechanisms, expanding programs to new settings and populations, and generating further evidence through registry and health services research.

## Discussion

### Principal Findings

This study provides in-depth insights into international implementation processes for evidence-based care programs for musculoskeletal disorders, with a primary focus on knee and hip OA and 1 related back pain program derived from an OA model. The findings were obtained from 12 expert interviews. Beyond documenting persistent challenges across preparation, initial implementation, and consolidation phases, our findings reveal a critical disconnect: while international networks and evidence-based models like GLA:D are widely valued, systematic knowledge exchange and joint solutions remain rare. In some instances, international cooperation even required additional coordination and administrative effort. Nonetheless, the possibility of adapting and translating existing program materials, rather than developing entirely new ones, was seen as a major advantage. Westerlund et al [16] highlight that implementation science encompasses a growing range of frameworks, models, and theories aimed at bridging the gap between knowledge generation and its application in health care practice [9]. Evidence suggests differing perceptions between researchers (knowledge producers) and policymakers (knowledge users) [8]. Knowledge producers tended to underestimate their influence on policy while overemphasizing barriers to implementation [8]. While opinions differ on whether this gap can be bridged by theoretical approaches in implementation science, it can be assumed that the application of theory is explicit and verifiable but might lead to problems being overlooked that are not addressed in theory. Applying common sense for implementations is often based on implicit assumptions and beliefs [13]. Nilsen underlines that implementation needs to be connected to “the real world of implementation practice” [13].

### Multiframework Interpretation

#### Connecting Knowledge, Barriers, and Implementation Practice

To situate the disconnect between available knowledge, perceived barriers, and actual implementation practice, we draw on 3 complementary implementation frameworks following Nilsen’s typology of (1) process models guiding the implementation, (2) determinant frameworks, classic theories, and implementation theories explaining determinants of implementation outcomes, and (3) frameworks evaluating implementation [13]. These frameworks did not guide data collection or coding but served to contextualize the generated findings.

#### Process Perspective: Knowledge-to-Action

The Knowledge-to-Action (KTA) Framework [10] is frequently used in different health care settings for the design, delivery, and evaluation of implementation activities [11]. The action cycle includes central, iterative phases that can be interpreted as conceptually corresponding to the phases presented in the inductively derived categories of our study. Our phase (1) preparation matches the KTA steps 1‐3 (problem identification, knowledge adaptation, barrier assessment), since experts described their motivations (equity, COVID-driven digitalization) and development tasks (cultural adaptation, train-the-trainer materials). Our phase (2) early implementation matches KTA steps 4‐5 (tailoring, execution), since regional pilots expanded through local champions and coordination gaps persisted. Phase (3) consolidation matches KTA steps 6‐7 (evaluation, sustainment), where registries enabled pre-post comparison, although short-term research funding threatened sustainability. The KTA cycle breaks down the phases into specific steps and shows in step 4 (tailoring implementation) that systematic cross-national knowledge exchange was rare, and this gap in the action cycle might prevent scale-up.

#### Determinant Perspective: Consolidated Framework for Implementation Research

Nilsen underlines that implementation needs to be connected to “the real world of implementation practice” [17]. One suitable framework for the determinant perspective is the Consolidated Framework for Implementation Research (CFIR), originally developed in 2009 [18] and revised in 2022 [19]. It identifies constructs influencing implementation success and effectiveness in 5 main areas (innovation, outer setting, inner setting, individuals, and implementation process), as well as 3 areas in the addendum (antecedent assessments, implementation outcomes, and innovation outcomes).

In the innovation domain, the evidence base of the programs needs to be mentioned (construct of innovation evidence base). The possibility of continuing to use existing evidence-based OA treatment programs and the possibility of adaptation to different (regional) contexts fit the construct of innovation adaptability. Ensuring the program’s advantage over other types of treatment (eg, conservative vs surgical treatment; digital vs analog vs blended care) fits the construct of innovation relative advantage. Regarding the outer setting domain, financing emerged as a dominant barrier across phases (construct financing). This prominence of financing across all implementation phases confirms previous implementation research identifying sustainable funding as one of the strongest determinants of successful implementation. Project funding ended post-efficacy proof, while reimbursement negotiations and license costs hindered rollout. Research costs are identified as a frequently mentioned hurdle to implementation [20]. This reflects the broader challenge of insufficient funding for implementation research in health care, where many projects support development but fail to ensure integration into routine care, a problem widely documented across different areas of the health care system [18]. Political support was mentioned as essential but slow (construct policies and law). Political challenges discussed in the interviews relate primarily to national reimbursement policies and regulatory frameworks. As described in the review by Gesulga et al [21], political support constitutes a major challenge when it comes to implementation.

In the inner setting domain, interviewees noted that structured communication channels significantly enhanced program satisfaction and quality, especially in the consolidation phase (construct communications).

In the individuals domain, it became clear that an initially well-connected team with knowledge from the field is particularly important (construct implementation team members). Interviewees emphasized the importance of motivation (construct motivation), technical expertise (eg, in project management, research, and therapy) (construct capability), and existing networks for effective implementation (construct opportunity).

In the implementation process domain**,** the gradual rollout from the regional to national level through local pioneers reflects the CFIR constructs (construct planning). Engaging stakeholders such as national opinion leaders, formally appointed implementation leads, and local pioneers among therapists accelerated awareness (construct engaging). Registry analyses, annual reports, and feedback loops informed adjustments to content, didactics, and quality assurance measures (construct reflecting and evaluating).

Our findings extend previous CFIR-based work by illustrating how the relevance of specific determinants changes across different implementation phases.

#### Evaluation Perspective: Reach, Effectiveness, Adoption, Implementation, Maintenance Framework

The RE-AIM (Reach, Effectiveness, Adoption, Implementation, Maintenance) Framework conceptualizes the impact on public health across its name-giving key dimensions, highlighting the importance of evaluating outcomes at the individual and organizational levels [22]. Interviewees prioritized reach (eg, patient access, offering programs in various cost systems), while digital formats addressed accessibility for rural/urban patients, and group training mitigated workforce shortages. Participants perceived improvements in precomparisons and postcomparisons and better preparation for surgery after the programs. Registries enabled cross-national comparisons, supporting policy advocacy from the participants’ point of view. Program adoption is hindered by limited therapist uptake, which is further challenged by heavy workloads, reimbursement gaps, and group logistics. Implementation was influenced by train-the-trainer fidelity. Regarding maintenance, recertification ensured consistency, although smaller practices struggled. Feedback loops (patient satisfaction, provider critiques) drove refinements such as recertification and didactics.

#### Comparison With Existing Guidelines

In 2019, the OA Research Society International published an international consensus guideline on priorities for the effective implementation of OA care, emphasizing, among other objectives, the need for standardized implementation approaches that align with international best practices [23]. Our findings suggest that several of these objectives remain only partially achieved, particularly regarding sustainable financing models (objective 3: operational funding for programs) and consistent reimbursement structures (objective 12: reimbursement of out-of-pocket costs for participants), as well as systematic implementation support and training for clinicians (objective 5: skills, confidence, and training of health care professionals). The experts perceived a persistence of implementation challenges since 2019 and attributed this to slow policy responses, unchanged or fragmented funding arrangements, and insufficient alignment between health system incentives and guideline recommendations.

#### Synthesis of the Interpretation

Taken together, the 3 frameworks shed light on complementary aspects of implementation. While the KTA framework helps us understand implementation as a dynamic process spanning various phases, CFIR explains the determinants that influence the success of implementation within these phases. RE-AIM complements this perspective by focusing on long-term adoption and maintenance. Our synthesis underscores Nilsen’s approach [13], because the combined application of these frameworks and guidelines enables a more comprehensive understanding of the implementation of evidence-based OA care programs in different health care systems.

### Limitations

This study represents an important exploratory step toward understanding the implementation of evidence-based OA treatment programs in different international contexts. However, some limitations must be considered.

By focusing on experts, we were able to extract subject-specific expertise regarding the practical implementation of OA programs. On the other hand, the exclusive reliance on expert interviews restricts the range of perspectives, particularly as patient views are therefore not included.

Regarding the selection of interviewees, the overrepresentation of experts affiliated with GLA:D-related programs may have influenced the thematic emphasis of the findings. In addition, the international sample was recruited through purposive and snowball sampling, which may have favored well-established professional networks and highly visible programs while excluding less prominent initiatives and perspectives.

Another limitation relates to the fact that most participants were directly involved in the development, implementation, or management of the programs under investigation. Their close engagement may have influenced the way implementation successes and challenges were described, potentially resulting in a more favorable assessment of the programs.

Although the interview guide covered the main aspects of implementation, it was not explicitly developed using a comprehensive implementation framework. Consequently, some contextual aspects, such as regional or federal policy influences, may not have been explored in equal depth.

By focusing exclusively on qualitative interviews, the study did not include methodological triangulation (eg, additional quantitative data). Consequently, the results could not be supported by multiple types of evidence.

Similarly, the findings were not externally validated through participants or additional stakeholder validation in the sense of member checking. While participant validation is not considered essential in all qualitative research traditions, its absence may have limited the opportunities to further enhance the confirmability of the findings.

Although purposive sampling allowed us to maximize variation in implementation experiences across countries and program types, transferability of the findings depends on similarities between health care systems, reimbursement structures, regulatory environments, and organizational contexts. The findings should therefore be interpreted in relation to the specific health care contexts in which the included programs were implemented.

Future research should address specific gaps identified in this study, including the cost-effectiveness of implementation strategies, the contextual adaptation of programs to diverse health system contexts, and implementation in resource-limited settings.

### Conclusion

This qualitative study explored facilitators and barriers influencing the international implementation of evidence-based nonsurgical OA programs from the perspective of experts (n=12) involved in established international programs.

Participants emphasized the importance of international collaboration, established program structures, and access to implementation experience. They highlighted the importance of stakeholder engagement, effective communication, and interdisciplinary collaboration throughout the implementation process. Furthermore, participants emphasized that context-appropriate combinations of digital, analog, and blended care models may improve accessibility, scalability, and long-term sustainability of OA programs. Fragmented reimbursement structures and short-term funding were consistently described as key barriers to sustainable implementation.

These insights provide practical guidance for researchers, clinicians, and policymakers seeking to implement evidence-based OA care programs across diverse health care systems while acknowledging the importance of adapting implementation strategies to local contexts.

## Supplementary material

10.2196/85941 Multimedia Appendix 1 Interview guide in English.

## Abbreviations

CFIR: Consolidated Framework for Implementation Research
COREQ: Consolidated Criteria for Reporting Qualitative Research
GLA:D: Good Life with osteoArthritis in Denmark
KTA: Knowledge-to-Action
OA: osteoarthritis
RE-AIM: Reach, Effectiveness, Adoption, Implementation, Maintenance
