# Supplementary material for: Barriers and Facilitators in the Implementation of Digital, Blended, and Analog Evidence-Based Osteoarthritis Care: International Qualitative Expert Interview Study
Source: JMIR Rehabil Assist Technol. 2026 Oct 23;13:e85941. doi: 10.2196/85941 (PMC13693861; doi:10.2196/85941)
Supplement: Supplementary file 1 [file rehab-v13-e85941-s001.pdf]

Interview guideline for the study "Verifying the transferability of the GLA:D® concept ('Good Life with OsteoArthritis in Denmark') to the context of the German health care system (Ü-GLAD))."

Introduction

Welcome and thank you for taking the time to participate in this interview.

I would like to begin by briefly introducing the Ü-GLAD project and the purpose of this interview. The aim of the project is to compile scientific evidence on the implementation of programs that use conservative treatment methods for knee and hip osteoarthritis. We further want to examine the transferability of these programs to the German health care system. By interviewing experts like you, we want to fill in the gaps in the scientific evidence on the program implementation and expand the existing knowledge.

Therefore, the purpose of this interview is to capture your experiences and expertise regarding the implementation of such programs for the conservative treatment of knee and hip osteoarthritis. During the interview I use the term “implemented” for all programs that can be demanded by patients with osteoarthritis in standard care. Further, I will only use the term osteoarthritis, while specifically referring to knee and hip osteoarthritis.

For international programs:

We will conduct further interviews with experts from other countries and with other programs. Therefore, we are specifically interested in your perspective on the implementation process in the country you were responsible for.

We are interested in your personal experiences, so there are no right or wrong answers. All your thoughts on the subject are important to us.

In turn, it is very important to us that you feel comfortable. Therefore, I would ask you to tell me, if you don’t feel comfortable with answering a question or are not able to. I will move to the next question then.

Do you have any questions about the content of this interview so far?

Do you have any questions about the study information and consent form that we sent you prior to the interview?

Do you agree with the interview being recorded by an audio device, so we can listen to it again? Thank you, I will turn on the recorder now.

Thank you very much for your participation in this interview and your consent to have the interview recorded. We now want to talk about your experience in implementing the program for the conservative treatment of osteoarthritis.

Subject area I: Context and background

- You are/were employed by/have done research on the [...] program. Please give a brief description of the program and its components.
    - What are the goals of the program?
    - How is the intervention itself structured?
    - What occupational groups are involved?
- How did you develop the program design of [...]?
    - Has there been a previous study on which the program [...] was based? In which country has the program been carried out?
        - Can you describe the study design to us?
- *If the interviewed person was involved in the program:* The program lists you as the person responsible for [...]. Please describe your role and the tasks related to this role.
- What changes did the program cause in the standard care of osteoarthritis in your country?
- How was osteoarthritis care provided in your country (before the program)?
    - Which care services for osteoarthritis required a prescription (e.g., physical therapy, pain medication)?
    - Which care service did not require a prescription?

    - What occupational groups were involved in providing this care?

Subject area II: Implementation of the program

- Please describe how the decision was made to implement the program in standard care.
    - Who initiated this decision? (Project staff*, health policy makers*, etc.).
- What adjustments did you have to make before implementation?
    - In case that the program was based on a program from a different country –How did you adapt the program to your country's health care system?
        - What adaptations have been necessary on a political-institutional level (economic, infrastructural, material, bureaucratic, legal)?
    - What challenges did you experience in the implementation process?
- What actions were taken in order to implement the program?
    - Can you explain the implementation process step by step?
    - How was the composition of the team responsible for the implementation process?
    - Have further adjustments been necessary after the initial implementation? If so, what kind of adjustments?
- What reactions did you get from health policy/providers/investors on implementing the program countrywide?
    - Did you get any negative reactions? What were the reasons for the concerns brought to you? To what extent could you relate/not relate to these concerns?

Subject area III: Experiences with/since implementation

- How is the reimbursement for trainers/ physiotherapists using the program performed?
    - Is there any additional remuneration?
    - How are further education costs for trainers/physiotherapists covered?
- Did the service providers face additional costs (e.g. purchase of new equipment, remodeling of practices) that needed to be covered? Who paid these costs?

- Who pays for the treatment costs incurred by patients that use the program? Are these costs covered by insurance or do patients have to pay for them out-of-pocket?
- What is you estimation about the additional workload of the program for trainers/physiotherapists in the care of osteoarthritis patients compared to the previous osteoarthritis treatment? Is there an additional effort for these people due to preparation and follow-up or supervision and education?
- What feedback have you received from participating physiotherapists since the start of the program regarding their motivation to participate? Has this feedback changed since the start of the program until now?
- What feedback have you received from participating physiotherapists regarding their satisfaction with the program since it has started? Has this feedback changed since the program began?
- What support do therapists get in order to incorporate the program into their practices?

Subject area IV: Own assessment & individual ideas for the future

- How satisfied are you with the current care structure in your country regarding the treatment of osteoarthritis?
    - Inquiry: What leads to this satisfaction/dissatisfaction?
- What should be different/better in the care structure? What changes would you like to see?
- How does the program [...] you are involved in/have been researching contribute to improving care of osteoarthritis?
- What advice would you give to a country planning to implement a conservative care program for osteoarthritis?

Subject area V: Conclusion

- I have asked all relevant questions. Do you have any additional topic that you would like to address?

- If you have any additional thoughts on the interview later on please feel free to send us a written supplement.
- Would you agree with us contacting you again in case that we have any follow-up questions after re-listening the interview (e.g., due to acoustic quality of the recording or regarding the content)?
